# Sex differences in brain protein expression and disease

**DOI:** 10.1038/s41591-023-02509-y

**Published:** 2023-08-31

**Authors:** Aliza P. Wingo, Yue Liu, Ekaterina S. Gerasimov, Selina M. Vattathil, Jiaqi Liu, David J. Cutler, Michael P. Epstein, Gabriëlla A. M. Blokland, Madhav Thambisetty, Juan C. Troncoso, Duc M. Duong, David A. Bennett, Allan I. Levey, Nicholas T. Seyfried, Thomas S. Wingo

**Affiliations:** 1 Veterans Affairs Atlanta Health Care System, Decatur, GA USA; 2 Department of Psychiatry, Emory University School of Medicine, Atlanta, GA USA; 3 Department of Neurology, Emory University School of Medicine, Atlanta, GA USA; 4 Department of Human Genetics, Emory University School of Medicine, Atlanta, GA USA; 5 Department of Psychiatry and Neuropsychology, Maastricht University School for Mental Health and Neuroscience, Maastricht, the Netherlands; 6 Clinical and Translational Neuroscience Section, Laboratory of Behavioral Neuroscience, National Institute on Aging, National Institutes of Health, Bethesda, MD USA; 7 Department of Pathology, Johns Hopkins School of Medicine, Baltimore, MD USA; 8 Department of Biochemistry, Emory University School of Medicine, Atlanta, GA USA; 9 Rush Alzheimer’s Disease Center, Rush University Medical Center, Chicago, IL USA; 10 Goizueta Alzheimer’s Disease Center, Emory University School of Medicine, Atlanta, GA USA

**Keywords:** Gene expression profiling, Alzheimer's disease, Gene regulation

## Abstract

Most complex human traits differ by sex, but we have limited insight into the underlying mechanisms. Here, we investigated the influence of biological sex on protein expression and its genetic regulation in 1,277 human brain proteomes. We found that 13.2% (1,354) of brain proteins had sex-differentiated abundance and 1.5% (150) of proteins had sex-biased protein quantitative trait loci (sb-pQTLs). Among genes with sex-biased expression, we found 67% concordance between sex-differentiated protein and transcript levels; however, sex effects on the genetic regulation of expression were more evident at the protein level. Considering 24 psychiatric, neurologic and brain morphologic traits, we found that an average of 25% of their putatively causal genes had sex-differentiated protein abundance and 12 putatively causal proteins had sb-pQTLs. Furthermore, integrating sex-specific pQTLs with sex-stratified genome-wide association studies of six psychiatric and neurologic conditions, we uncovered another 23 proteins contributing to these traits in one sex but not the other. Together, these findings begin to provide insights into mechanisms underlying sex differences in brain protein expression and disease.

## Abstract (web summary)

Analyses of 1,277 human brain proteomes reveal the extent of sex differences in the brain and identify genes associated with neuropsychiatric traits that have differential regulation between males and females.

## Main

Differences between females and males abound among human traits and disease. For instance, the prevalence of common psychiatric and neurologic conditions such as major depressive disorder^1^, schizophrenia^2^, Parkinson’s disease^3^ and Alzheimer’s disease (AD)^4^ differ by sex. Even what constitutes disease risk may differ by sex. For instance, females have significantly higher risk for myocardial infarction at lower systolic blood pressure than males^5^. Recent genetic studies have also found genetic risks differ by sex for psychiatric and neurologic conditions^6,7^. Underlying reasons complex traits differ by sex may stem from many factors, including physiologic, genetic and environmental^8^.

Differences in gene expression by sex have been observed in human brain across the major developmental stages (prenatal, early childhood, puberty and adulthood)^9^ and in splicing^10^. Reasons for sex-biased gene expression for some autosomal genes may be related to the presence of androgen or estrogen hormone response elements^11^. Sex-biased gene expression likely contributes to differences in prevalence or manifestation of psychiatric and neurologic conditions^12–17^. Previous studies of sex-biased genetic regulation of gene expression have focused on transcripts in up to 150 post-mortem brain tissues^12,13^. These studies relied on hard-to-obtain post-mortem brain tissues, making large-scale studies challenging. Thus, while these studies provided valuable insights, larger studies and ones that examine both transcript and protein expression are needed. No study, however, has examined sex differences in brain protein expression, to our knowledge. Directly testing the effect of sex on protein expression is important because of the low correlation between messenger RNA (mRNA) and protein levels^18–21^, possibly due to layers of post-transcriptional regulation that are also likely influenced by sex.

**Fig. 1:**
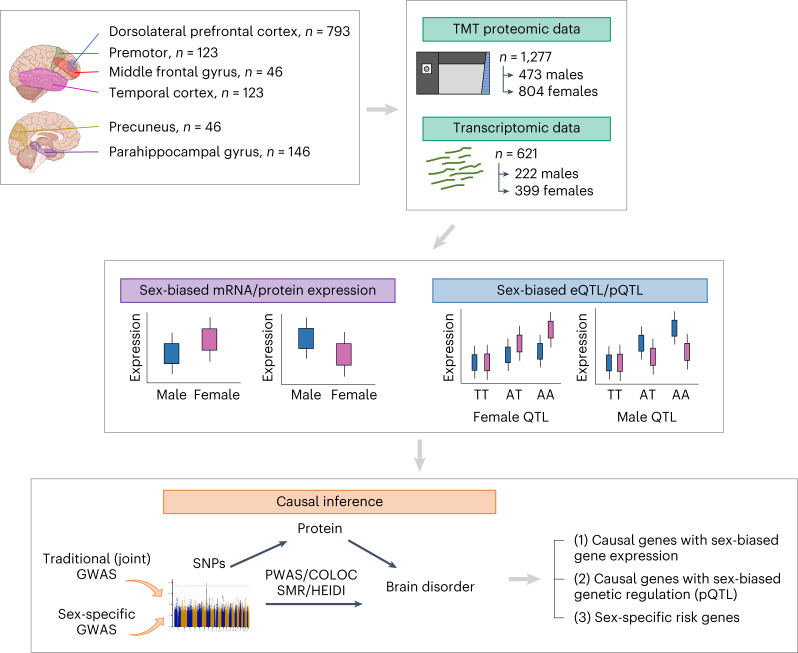
Summary of main analyses. We investigated sex differences in protein expression and its genetic regulation using brain proteomic and genetic data. Next, we compared effects of sex on gene expression and its genetic regulation at both the mRNA and protein levels using genetic and brain transcriptomic and proteomic data. Subsequently, we examined the intersection between psychiatric and neurologic causal genes and genes with sex-biased protein expression or sb-pQTLs. Finally, we integrated sex-stratified GWAS with pQTLs to identify sex-specific causal genes in psychiatric and neurologic disorders. TMT, tandem mass tag.

To address these knowledge gaps, we investigated the influence of biological sex on protein abundance and its genetic regulation using 1,277 human brain proteomes (Fig. 1). Next, we compared effects of sex on gene expression and its genetic regulation at the transcript and protein levels using data from 621 human brain transcriptomes whose donors were a subset of the donors of the brain proteomes. Finally, we investigated connections between sex-differentiated brain protein abundance and a range of psychiatric and neurologic conditions (Fig. 1). Collectively, these results shed light on the effects of sex on gene expression at both the transcript and protein levels and identify new molecular mechanisms underlying the role of sex in brain disease.

## Results

### Sex differences in brain protein abundance

Deep brain proteomes from 1,277 donors of European ancestry were generated from six brain regions with 62% (793 of 1,277) from the dorsolateral prefrontal cortex (dPFC; Supplementary Table 1). Sex was inferred from X-chromosome genotyping and was consistent with self-reported sex for all donors. After quality control, 10,198 proteins were considered, of which 371 (or 3.6%) were encoded by genes on the X chromosome. Before testing for sex-biased expression, the effects of protein sequencing batch, post-mortem interval, donor age and clinical diagnosis were estimated and removed using linear regression, and surrogate variable analysis (SVA) was used to infer hidden technical or biological factors that may influence brain protein levels, including cell-type composition. To identify genes with sex-biased expression, we fit a linear regression model with protein expression as the outcome, sex as the independent variable and surrogate variables (SVs) as covariates in each brain region separately.

**Extended Data Fig. 1:**
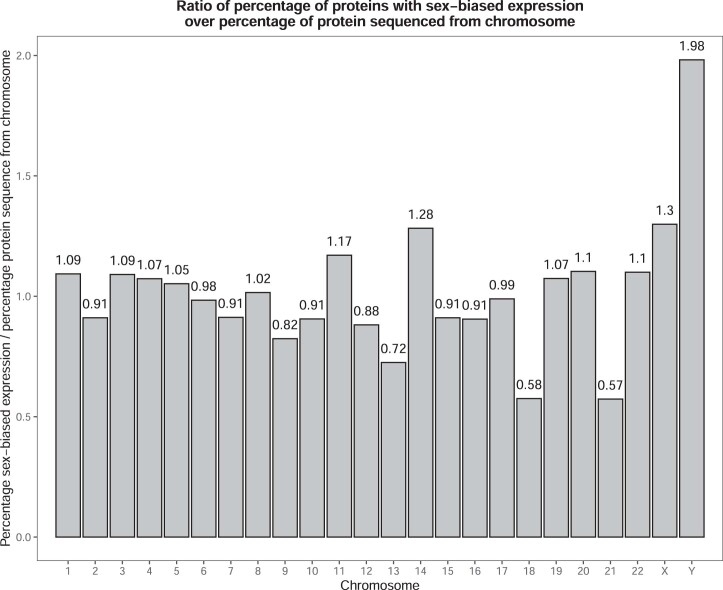
Proteins with sex-biased expression per chromosome. Ratio of percent of proteins with sex-differentiated abundance over percent of proteins profiled in the brain proteomes for each chromosome. This enables comparison of the number of genes with sex-differentiated protein abundance across the chromosomes, taking into consideration the number of genes encoded on each chromosome and the number of these proteins profiled in the brain proteomes.

Among 10,198 measured proteins, 1,239 differed by sex in the dPFC at the false discovery rate (FDR) <0.05 and, of these, 4.8% were encoded by genes on the X chromosome (Supplementary Table 2). Among the 1,239 proteins, 51% had higher expression in females and 49% had higher expression in males (Supplementary Table 2). As expected, the sex chromosomes had the highest proportions of genes with sex-biased expression, whereas the autosomes had roughly similar proportions (Extended Data Fig. 1). Since different brain regions may have different cell-type composition and biological functions, we tested for sex-biased expression in five additional regions: parahippocampal gyrus, temporal cortex, premotor, precuneus and middle frontal gyrus (Supplementary Table 2). Within each region, about half of the proteins had higher abundance in males, while the other half had higher abundance in females at FDR < 0.05 (Supplementary Tables 2 and 3), which was consistent with findings from the dPFC.

To identify proteins with sex-biased protein expression across multiple brain regions, we used multivariate adaptive shrinkage (MASH)^22^ to perform a meta-analysis for each protein across all measured brain regions and estimated a local false sign rate (LFSR) for each protein. LFSR is analogous to the FDR but more stringent^22^. Sex-biased gene expression was observed in each of the six brain regions examined (Supplementary Tables 2,3). We found 13.2% (1,354 of 10,198) proteins with sex-biased protein expression in at least one brain region at MASH LFSR < 0.05 and, among those, 4.7% were encoded by genes on the X chromosome (Supplementary Tables 2 and 3).

### Sex differences in genetic control of brain protein abundance

**Fig. 2:**
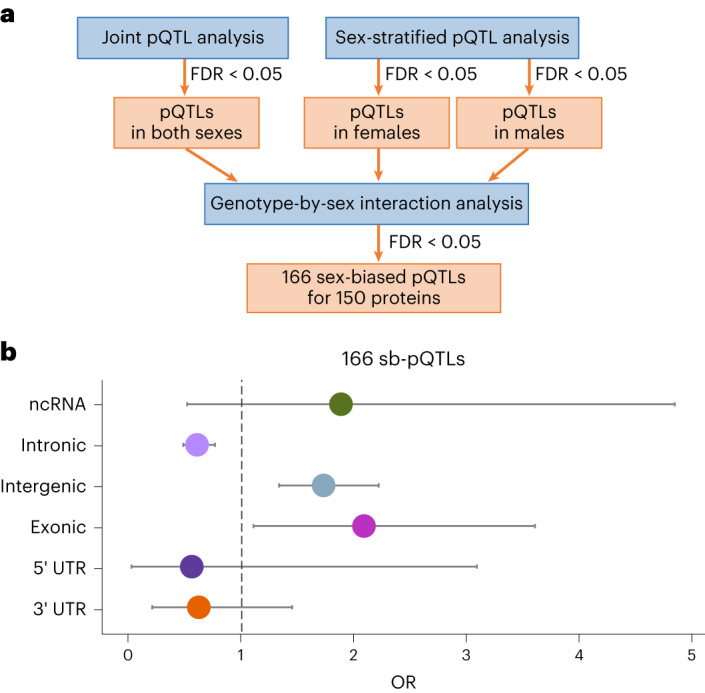
Sex-biased pQTLs. **a**, Operational definition of sb-pQTLs: an SNP needs to meet both of the following two criteria to be declared a sb-pQTL. First, it is a pQTL in males or females or both at FDR < 0.05. Second, it has a significant genotype-by-sex interaction in protein expression at FDR < 0.05. We identified 166 index sb-pQTLs corresponding to 150 unique proteins in human brain. **b**, Genomic site-type enrichment of 166 index sb-pQTLs. Data are presented as OR ± 95% confidence interval. Fisher’s exact test was used to calculate the ORs. Error bars reflect a 95% confidence interval. ncRNA, non-coding RNA; UTR, untranslated region.

We next investigated whether there is a difference in the genetic regulation of protein abundance between females and males by performing sex-biased protein quantitative trait locus (sb-pQTL) analysis in the dPFC, which is the brain region with the largest sample size with both genetic and proteomic data (*n* = 716). We examined proteins encoded by genes on the autosomes and the X chromosome. For the latter, we coded the number of minor alleles as 0, 1 or 2 for females and 0 or 2 for males who are hemizygous for the X chromosome. To identify proteins with sb-pQTL, we performed a two-stage analysis (Fig. 2a). The first stage comprehensively identified pQTLs and the second stage tested significant pQTLs for interaction with sex. To comprehensively identify pQTLs, we performed a pQTL analysis jointly in males and females, and in each sex separately. For each pQTL analysis, we adjusted for SVs and genetic principal components, and sex in the joint analysis. pQTLs were defined as single nucleotide polymorphisms (SNPs) that have an association with proteins at FDR < 0.05. In the second stage, pQTLs were tested for genotype-by-sex interaction, adjusting for the SVs and genetic principal components. sb-pQTLs were defined as pQTLs that have a significant genotype-by-sex interaction at FDR < 0.05 (Fig. 2a).

**Extended Data Fig. 2:**
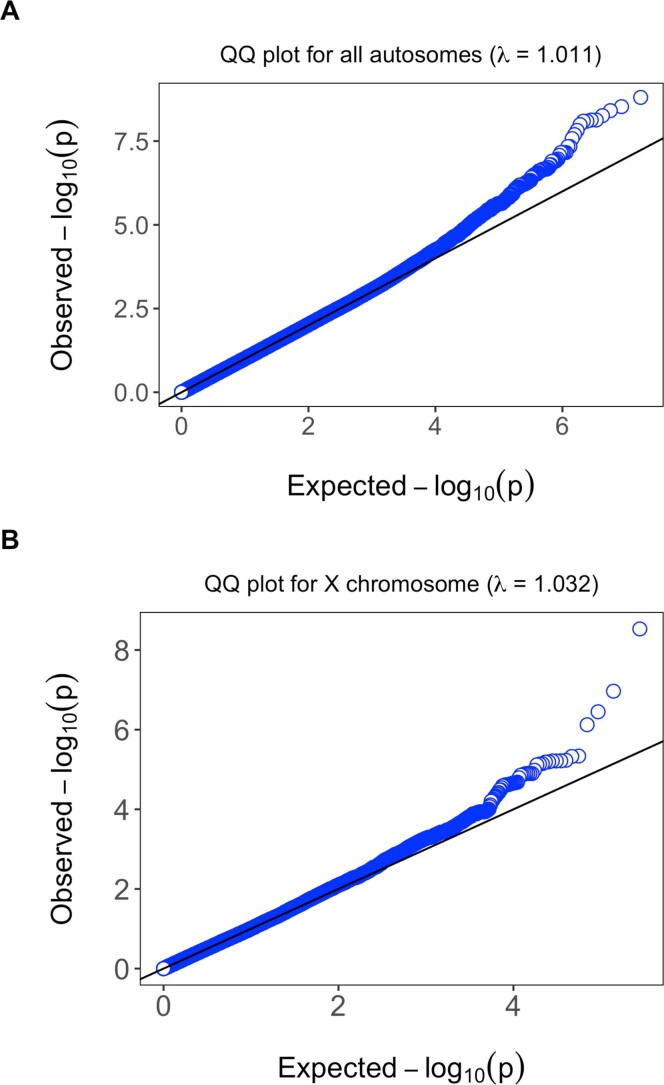
Quantile–quantile (QQ) plots. QQ plots for the *P* values of the genotype-by-sex interaction term in the regression model *protein ~ sex* + *SNP* *+* *sex* *×* *SNP* + *56 SVs* + *10 PCs* for all autosomes (**a**) and the X chromosome (**b**). The genomic inflation factor lambda was 1.011 for all chromosomes and 1.032 for the X chromosome.

There were 1,036,025 pQTLs identified in the first stage and 1,171 sb-pQTLs identified in the second stage for 150 unique proteins. Linkage disequilibrium clumping of the 1,171 sb-pQTLs yielded 166 index sb-pQTLs (at *r*^2^ < 0.5) or 154 independent sb-pQTLs (at *r*^2^ < 0.1), both of which corresponded to 150 unique proteins. For the 166 index sb-pQTLs, 48% had a positive beta coefficient and 52% had a negative beta coefficient for the genotype-by-sex interaction term (Supplementary Table 4). The quantile–quantile plot for the *P* values of the genotype-by-sex interaction term in the regression modeling for all the autosomes (Extended Data Fig. 2a) and the X chromosome (Extended Data Fig. 2b) showed no evidence of inflation. Of the index sb-pQTLs, 5.4% were pQTLs in both sexes with concordant direction of association, 2.4% were pQTLs in both sexes with discordant direction of association, 51.2% were pQTLs in males only and 37.9% were pQTLs in females only (Supplementary Tables 4–6). These sb-pQTLs were enriched for intergenic (odds ratio (OR) = 1.73; *P* = 4.2 × 10^−5^) and exonic SNPs (OR = 2.1; *P* = 0.01) and depleted for intronic SNPs (OR = 0.6; *P* = 2.2 × 10^−5^; Fig. 2b). Among the 150 proteins with sb-pQTLs, nine were encoded by genes on the X chromosome and none were in the pseudo-autosomal region of the X chromosome (Supplementary Table 7).

We found that 17% of the proteins with sb-pQTLs (25 of 150) also had sex-differentiated protein abundance (Supplementary Table 8). Our findings at the protein level are comparable with published findings at the transcript level, in which 14% of the sex-biased expression quantitative trait locus (sb-eQTL) transcripts also had sex-biased mRNA expression^12^.

To determine the internal replication rate (*π*_1_) for sb-pQTLs, we used the Religious Orders Study (ROS)/Rush Memory and Aging Project (MAP) dataset as the discovery sample (*n* = 565) and the Banner dataset (*n* = 151) as the replication sample. The *π*_1_ replication statistic estimates the rate of sb-pQTLs identified in the discovery dataset that are sb-pQTLs in the replication dataset. The internal replication rate for sb-pQTLs was 0.52, which implies that 52% of the sb-pQTLs identified in the discovery sample were also sb-pQTLs in the replication sample. For context, in the largest sb-eQTL study to date, the most sb-eQTLs were identified in breast tissue and the internal replication rate for sb-eQTLs in breast tissue was 0.28 (ref. ^12^).

To test whether environmental factors could explain the modifying effect of sex on genetic regulation of protein expression for the identified 150 proteins with sb-pQTLs, we examined the environmental factors present in our dataset, including lifetime alcohol use, smoking, comorbid medical conditions and education. We found a significant difference between males and females for education, alcohol use and smoking but not for comorbid medical conditions. Next, we determined whether there was a significant SNP-by-environment term in the regression model ‘protein ~ SNP + environment + SNP × environment + SVs + 10 principal components’ for the environment factor of education, alcohol use and smoking, respectively. We used the *P-*value threshold of 5.6 × 10^−5^ since it was the *P-*value threshold for FDR < 0.05 for the sb-pQTL analysis. We found the SNP-by-environment term to be significant in one SNP for education and five SNPs for lifetime alcohol use among the 166 index sb-pQTLs. Then we tested whether the genetic interaction with sex remained significant when considering a sex-by-environment term for these six SNPs using the regression model ‘protein ~ SNP + sex + environment + SNP × sex + SNP *×* environment + SVs + 10 principal components’. Among these six SNPs, four continued to have a significant SNP × sex term (education and alcohol use) and two no longer had a significant SNP × sex term (alcohol use; Supplementary Table 9). Thus, among the 166 index sb-pQTLs, only two may be potentially affected by the difference in lifetime alcohol use between males and females, lending confidence to the sb-pQTL findings.

### Sex-biased expression at both the mRNA and protein levels

We next examined genes with sex-differentiated expression in human brain at both the mRNA and protein levels. First, we identified sex-differentiated mRNA expression using transcriptomic profiles from the dPFC of 621 donors of European ancestry (Supplementary Table 1). After quality control and normalization, 15,582 mRNAs were included in the analysis and 500 (3.2%) mRNAs were encoded by genes on the X chromosome. Before testing for sex-biased expression, the effects of batch, RNA quality, post-mortem interval, donor age and clinical diagnosis were estimated and removed using linear regression, and SVA was used to infer hidden technical and biological variables. To estimate the effect of sex on brain mRNA expression, we fit a linear regression model with mRNA levels as the outcome, sex as the independent variable and SVs as covariates. We found that 4,279 (27.5%) mRNAs had different expression levels between males and females at FDR < 0.05 and, among these, 226 mRNAs (or 5.3%) were encoded by genes located on the X chromosome (Supplementary Table 10).

Interestingly, there were 498 (5.5%) genes with sex-differentiated expression at both the mRNA and protein levels among the 9,080 genes measured in both the transcriptomic and proteomic profiles (Supplementary Table 11). Among these 498 genes, 76.1% had concordant sex-biased expression for mRNA and protein. The replication rate (*π*_1_) of sex-biased expression between proteins and mRNAs was 0.67. Genes with discordant sex-biased expression between mRNA and protein were enriched for proteins involved in axonal growth cone (adjusted *P* = 0.013). Genes with concordant sex-biased expression between mRNA and protein were enriched for proteins involved in cellular morphology, cell adhesion, actin filament organization, initiation of translation and branched-chain amino acid degradation (Supplementary Table 12).

### Comparing sex-biased genetic regulation of mRNAs versus proteins

To understand the shared and distinct sex effects on genetic regulation of brain proteins and mRNAs, we compared sb-pQTLs with sb-eQTLs. First, we identified sb-eQTLs using the same two-stage approach as was used to identify sb-pQTLs (Fig. 2) in 589 donors of European ancestry with genetic and transcriptomic data from the dPFC (Supplementary Table 1). We observed no sb-eQTLs at FDR < 0.05 or FDR < 0.1. Relaxing the threshold to FDR < 0.2, there were 2,336 *suggestive* index sb-eQTLs, which corresponded to 1,834 unique mRNAs (Supplementary Table 13). Surprisingly, using the threshold of FDR *P* < 0.2 to define sb-QTLs, the replication rate *π*_1_ between sb-pQTLs and sb-eQTLs was 0 despite an internal sb-pQTL replication rate of 0.53.

We performed several internal and external checks of our sb-eQTL findings. First, our sb-eQTL findings are in line with those from a prior study that detected sb-eQTLs in brain only at FDR < 0.25 but not at a lower FDR threshold^12^. Second, we found the *π*_1_ replication rate between our eQTLs and a large published eQTL study^23^ to be 0.96, lending confidence in our QTL analysis. Third, we investigated whether higher interindividual variations in mRNA levels or sample sizes (*n* = 716 for sb-pQTL and *n* = 589 for sb-eQTL analysis) may partially drive the difference in sb-eQTLs and sb-pQTLs. To that end, we examined the number of pQTLs, eQTLs, sb-pQTLs and sb-eQTLs in two scenarios: (1) among all genes profiled in the proteomes and transcriptomes, respectively; and (2) only among genes profiled in both the transcriptomes and proteomes (*n* = 8,009 genes). Interestingly, we found more eQTLs than pQTLs in both scenarios and in both sexes together or in either sex alone at FDR < 0.05 (Supplementary Table 14). Focusing on sb-QTLs, we found comparable numbers of sb-eQTLs and sb-pQTLs at FDR *P* < 0.2 in both scenarios; however, there were more sb-pQTLs than sb-eQTLs at more stringent FDR thresholds for defining sb-QTLs (Supplementary Table 15). Together, these findings suggest that the interindividual variations in mRNA levels and sample size did not explain the difference in the number of sb-eQTL and sb-pQTLs.

### Putative causal genes with sex-biased protein expression

We asked whether any of the genes with sex-biased expression identified here are also causal genes in 24 psychiatric, neurologic and brain morphologic traits found in our recently published study^20^. In that work, we identified brain proteins that are consistent with a causal role in those traits by integrating 720 reference human brain proteomes with genome-wide association study (GWAS) results for each trait using multiple complementary approaches. These include proteome-wide association study (PWAS) using FUSION^24^, Mendelian randomization using SMR^25^ and colocalization analysis using COLOC^26^ to identify proteins with highest level of evidence for a causal role in each trait. For brevity, we refer to the identified 651 gene–protein pairs as causal genes or causal proteins recognizing that their causal role needs to be validated in model systems.

**Fig. 3:**
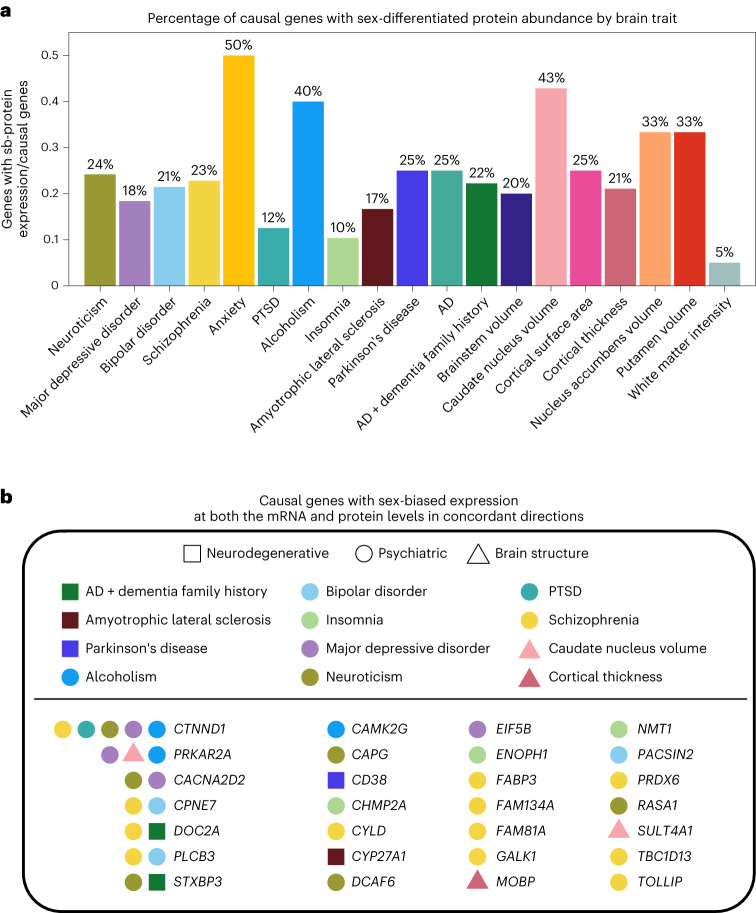
Causal genes in psychiatric, neurologic and brain morphologic traits with sex-biased protein abundance. **a**, Percentage of causal genes with sex-biased protein abundance among the identified causal genes for each brain trait. **b**, Causal genes with sex-biased expression at both the mRNA and protein levels in concordant directions (*n* = 28). Full results are in Supplementary Table 17. PTSD, post-traumatic stress disorder.

We found 97 causal genes with sex-biased protein expression by intersecting the 651 causal genes identified in the previous work with the 1,354 proteins with sex-biased expression identified in the current work (Supplementary Table 16). Moreover, we found that 25% of the causal genes in brain traits, on average, had sex-biased protein expression (range 5–50%; Fig. 3a). Furthermore, among these causal genes, 33 had sex-biased expression at both the brain mRNA and protein levels and 28 had concordant directions of sex-biased expression at the mRNA and protein levels (Fig. 3b and Supplementary Table 17). For instance, *CTNND1* is a causal gene in four different psychiatric disorders (major depression, schizophrenia, post-traumatic stress disorder and problematic alcohol use) and neuroticism (a personality trait that is prone to experiencing negative emotions) and had sex-biased expression at both the transcript and protein levels in concordant directions (Fig. 3b).

### Putative causal genes having sex-biased genetic regulation

To understand the role sex-biased genetic regulation of protein abundance may have on disease, we used two approaches designed to circumvent the limited power and small number of the available sex-stratified GWAS results.

**Fig. 4:**
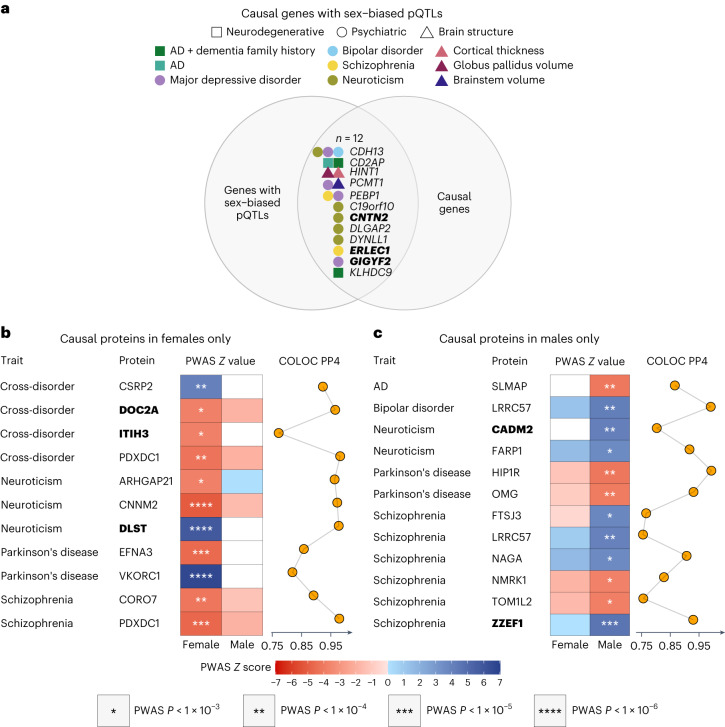
Sex-specific causal genes and proteins. **a**, Causal genes with sb-pQTLs (*n* = 12). Among these, three also had sex-biased protein expression: *CNTN2*, *ERLEC1* and *GIGYF2* (in bold). Detailed results are in Supplementary Table 18. **b**, Causal proteins in females only (*n* = 11). Among these 11 proteins, three also had sex-biased protein expression: DOC2A, ITIH3 and DLST (in bold). **c**, Causal proteins in males only (*n* = 12). Of these 12, two also had sex-biased protein expression: CADM2 and ZZEF1 (in bold). Cross-disorder refers to major depression, bipolar disorder and schizophrenia. Asterisks indicate significant *P* values in the sex-specific PWAS. Detailed results are in Supplementary Table 20.

In the first approach, we intersected 651 causal genes for psychiatric, neurologic and brain morphologic traits identified by the above-described work^20^ with 150 genes with sb-pQTLs identified in the current work. The intersection yielded 12 causal genes with sb-pQTLs (Fig. 4a and Supplementary Table 18a). To test the possibility of multiple independent causal variants within each of the identified genes, we performed Sum of Single Effects (SuSiE) regression^27^ for the 12 genes and did not find evidence for more than one causal variant per gene. These 12 genes influence four psychiatric, one neurologic and three brain structural traits, and 5 of 12 influence multiple traits (Fig. 4a). Notably, among these 12 causal genes, three also had sex-biased protein abundance: *ERLEC1*, *CNTN2* and *GIGYF2* (Fig. 4a and Supplementary Table 18b).

In the second approach, we performed sex-stratified PWAS and colocalization analysis in each sex separately to identify proteins with evidence consistent with a causal role in one sex but not in the other for six psychiatric and neurologic traits with available sex-stratified GWAS results^7,28,29^ (Supplementary Table 19a). Again, we refer to these as causal proteins for brevity. We defined sex-specific causal proteins as being significant in the PWAS in one sex (FDR *P* < 0.05) and having evidence of colocalization in that sex (posterior probability for hypothesis 4 (PP4) > 0.75) but not significant in the PWAS of the other sex (*P* > 0.05). Here, colocalization refers to colocalization of the genetic variants associated with the protein and trait of interest. We found 23 sex-specific causal proteins, with 11 for females only and 12 for males only (Fig. 4b,c, Tables 1 and 2 and Supplementary Table 20).

**Table 1:** Proteins consistent with a causal role in females only identified by PWAS and colocalization analyses using sex-stratified proteomic data and GWAS results (*n* = 11)

		PWAS and COLOC results in females				PWAS results in males		Published GWAS results		
Protein	Trait	PWAS Z	PWAS *p*	PWAS FDR *p*	COLOC PP4	PWAS Z	PWAS *p*	Best GWAS SNP	SNP×sex interaction *p*	suggestive sex-bias (interaction *p* < 0.05)
CSRP2	Cross-disorder	4.1	5.1 ✕ 10^−5^	1.8 ✕ 10^−2^	0.92	.	.	rs7302529	1.6 ✕ 10^−7^	yes
DOC2A	Cross-disorder	−3.7	1.8 ✕ 10^−4^	4.0 ✕ 10^−2^	0.97	−1.9	0.06	rs11150576	2.1 ✕ 10^−2^	yes
ITIH3	Cross-disorder	−3.8	1.6 ✕ 10^−4^	4.0 ✕ 10^−2^	0.77	.	.	rs2256332	2.3 ✕ 10^−1^	
PDXDC1	Cross-disorder	−4.2	2.7 ✕ 10^−5^	1.6 ✕ 10^−2^	0.98	−1.9	0.05	rs3751877	2.3 ✕ 10^−2^	yes
ARHGAP21	Neuroticism	−3.7	1.8 ✕ 10^−4^	2.4 ✕ 10^−2^	0.96	0.1	0.92	rs2152432	8.6 ✕ 10^−3^	yes
CNNM2	Neuroticism	−5.1	3.8 ✕ 10^−7^	2.3 ✕ 10^−4^	0.97	−1.5	0.12	rs732998	1.0 ✕ 10^−2^	yes
DLST	Neuroticism	5.8	6.3 ✕ 10^−9^	7.7 ✕ 10^−6^	0.98	.	.	rs8022046	1.7 ✕ 10^−1^	
EFNA3	Parkinson’s disease	−4.6	5.0 ✕ 10^−6^	1.4 ✕ 10^−3^	0.86	.	.	rs12743272	2.7 ✕ 10^−1^	
VKORC1	Parkinson’s disease	6.6	3.9 ✕ 10^−11^	4.7 ✕ 10^−8^	0.82	.	.	rs4889603	2.1 ✕ 10^−2^	yes
CORO7	Schizophrenia	−4.1	3.5 ✕ 10^−5^	1.4 ✕ 10^−2^	0.89	−1.3	0.21	rs3747584	1.9 ✕ 10^−2^	yes
PDXDC1	Schizophrenia	−4.6	3.8 ✕ 10^−6^	3.2 ✕ 10^−3^	0.98	−1.9	0.06	rs3751877	3.5 ✕ 10^−3^	yes

Multiple testing was adjusted with FDR. Evidence for sex bias at the genetic level for these proteins was based on published GWAS SNP-by-sex interaction *P* value. Full results are in Supplementary Table 20.

Proteins with missing values for PWAS *Z* and *P* value in one sex were those not included in the PWAS in that sex because the protein heritability estimate was not significant in that sex (that is, heritability *P* > 0.01) and the PWAS can only be performed on heritable proteins. PWAS FDR *P* value was adjusted for all proteins included in the sex-specific PWAS. Cross-disorder refers to cross-disorders among schizophrenia, bipolar disorder and major depression. Detailed results are in Supplementary Table 20.

**Table 2:** Proteins consistent with a causal role in males only identified by PWAS and colocalization analyses using sex-stratified proteomic data and GWAS results (*n* = 12)

		PWAS and COLOC results in males				PWAS results in females		Published GWAS results		
Protein	Trait	PWAS Z	PWAS *p*	PWAS FDR *p*	COLOC PP4	PWAS Z	PWAS p	Best GWAS SNP	SNP×sex interaction *p*	suggestive sex-bias (interaction *p* < 0.05)
SLMAP	AD	−4.1	4.4 ✕ 10^−5^	3.9 ✕ 10^−2^	0.87	.	.	rs266837	4.4 ✕ 10^−3^	yes
LRRC57	Bipolar disorder	4.1	3.9 ✕ 10^−5^	2.3 ✕ 10^−2^	0.99	1.3	0.21	rs4924687	1.5 ✕ 10^−3^	yes
CADM2	Neuroticism	4.1	4.3 ✕ 10^−5^	2.5 ✕ 10^−2^	0.8	.	.	rs1900916	1.8 ✕ 10^−1^	
FARP1	Neuroticism	3.6	3.5 ✕ 10^−4^	5.0 ✕ 10^−2^	0.92	1.5	0.13	rs2274051	1.6 ✕ 10^−1^	
HIP1R	Parkinson’s disease	−4.1	4.2 ✕ 10^−5^	9.3 ✕ 10^−3^	0.99	−1.2	0.23	rs11060180	3.9 ✕ 10^−1^	
OMG	Parkinson’s disease	−4.0	7.4 ✕ 10^−5^	1.2 ✕ 10^−2^	0.93	−0.9	0.36	rs11080149	4.0 ✕ 10^−2^	yes
FTSJ3	Schizophrenia	3.7	1.9 ✕ 10^−4^	2.5 ✕ 10^−2^	0.77	−0.5	0.63	rs1062791	6.4 ✕ 10^–3^	yes
LRRC57	Schizophrenia	4.0	6.4 ✕ 10^−5^	1.4 ✕ 10^−2^	0.76	0.9	0.38	rs2412709	8.7 ✕ 10^−2^	
NAGA	Schizophrenia	3.6	2.9 ✕ 10^−4^	3.5 ✕ 10^−2^	0.91	1.6	0.11	rs1023500	1.6 ✕ 10^−1^	
NMRK1	Schizophrenia	−3.7	2.0 ✕ 10^−4^	2.6 ✕ 10^−2^	0.83	−1.8	0.07	rs3780178	4.0 ✕ 10^−2^	yes
TOM1L2	Schizophrenia	−3.8	1.4 ✕ 10^−4^	2.2 ✕ 10^−2^	0.76	−1.6	0.11	rs4925133	3.7 ✕ 10^−1^	
ZZEF1	Schizophrenia	4.5	5.8 ✕ 10^−6^	1.7 ✕ 10^−3^	0.93	0.0	1	rs10521129	9.0 ✕ 10^−3^	yes

Multiple testing was adjusted with FDR. Evidence for sex bias at the genetic level for these proteins was based on published GWAS SNP-by-sex interaction *P* value. Full results are in Supplementary Table 20.

Proteins with missing values for PWAS *Z* and *P* value in one sex were those not included in the PWAS in that sex because the protein heritability estimate was not significant in that sex (that is, heritability *P* > 0.01) and the PWAS can only be performed on heritable proteins. PWAS FDR *P* value was adjusted for all proteins included in the sex-specific PWAS. Cross-disorder refers to cross-disorders among schizophrenia, bipolar disorder and major depression. Detailed results are in Supplementary Table 20.

To determine whether the identified sex-specific causal proteins show sex-specific genetic risk, we asked whether the genetic interaction with sex in the corresponding GWAS was nominally significant for the considered trait for the sites that were most significantly associated with the trait at the locus. There were 14 of 23 sex-specific risk proteins (61%) with suggestive evidence for sex-biased genetic risk (Tables 1 and 2 and Supplementary Table 20). We note that the sex-specific GWAS were limited in number and power compared with the joint GWAS (Supplementary Table 19a,b), making direct comparisons between the two approaches infeasible. Together, the 35 (12 and 23) identified sex-biased causal proteins are promising targets for sex-aware mechanistic studies for the 24 considered psychiatric, neurologic and brain morphologic traits.

## Discussion

We examined effects of biological sex on brain protein expression and disease at both the mRNA and protein levels. We found that approximately 27% of the mRNAs and 13% of the proteins had sex-differentiated expression in the brain. Furthermore, we found that only 5.5% of genes had sex-biased expression at both the transcript and protein levels, and 76% of these had concordant directions of sex difference. Next, we examined sex effects on the genetic regulation of gene expression and identified 150 proteins with sex-biased genetic regulation. To understand the relevance of our findings in brain health, we intersected our findings of sex-biased protein expression and genetic regulation of brain proteins with previously identified causal proteins for 24 psychiatric, neurologic and brain morphologic traits^20^. On average, we found that 25% of these causal proteins had sex-biased protein abundance and 12 causal proteins had sb-pQTLs. Furthermore, we integrated sex-specific GWAS with sex-specific brain protein data for six psychiatric and neurologic traits and identified 23 proteins consistent with a causal role in these conditions in one sex but not the other. Notably, 14 of 23 (61%) of these sex-specific causal proteins had suggestive evidence of having sex bias at the GWAS level despite the limited sample size and power of the published sex-stratified GWAS. Together, these results illuminate the effects of sex on brain health and lay a foundation for future sex-aware mechanistic studies of psychiatric and neurologic disorders.

To date, studies of sex effects on psychiatric and neurologic diseases have focused on brain transcriptome^15–17,30–32^. Consistent with the prominent role of the synapses and inflammation in sex-specific transcriptomic studies of depression^16^, our results at the protein level support the role of synaptic formation and immune function in sex-specific risk for depression. In particular, among the four causal genes with sb-pQTLs we found for depression, cadherin 13 (*CDH13*) regulates GABAergic neurons, axon guidance and synaptic formation^33,34^. Moreover, among the 18 depression causal genes with sex-biased protein expression that we identified, *GGH* and *PRKAR2A* are implicated in immune response^35–37^. In schizophrenia, a study of human brain transcriptomes found enrichment of gene coexpression modules with the sex-by-diagnosis differential mRNAs, and these modules contained genes enriched in neural development^15^. In line with these results, among the two schizophrenia causal genes with sb-pQTLs we identified, *PEBP1* is involved in neural development^38^. In sex-specific studies of alcoholism, a recent review of alcohol consumption studies using rodent models highlighted neuroimmune processes as a key emerging feature in sex differences in alcohol consumption^30^. In accordance with these observations, among the seven alcoholism causal genes with sex difference in brain protein expression that we identified, *LGALS3* is a driver of macrophage and microglia activation and has been implicated in neuroinflammation^39–41^. In AD, both human and mouse studies using brain transcriptomic data also observed the prominent role of microglial and inflammatory mechanisms in sex differences in AD^31,32^. In agreement with these results, among the eight AD causal genes with either sex-biased expression or genetic regulation of protein expression that we identified, half of them are involved in immune function: *CD2AP* facilitates recognition of antigen by T cells^42^; *SLMAP* participates in T cell receptor signaling^43^; *ADAM10* regulates cytokine levels in activated microglia^44^; and *STXBP3* is involved in immune function^45,46^.

An interesting facet of our results is that we did not find sb-eQTLs at FDR < 0.1 despite comparable sample sizes for the sb-eQTL and sb-pQTL analyses. We performed several verifications of our sb-eQTL findings and their results excluded sample size or interindividual variations in mRNA levels as potential explanations for the difference in significant sb-eQTLs and sb-pQTLs. We note that our sb-eQTL findings are consistent with those in published work that could only identify sb-eQTLs at FDR < 0.25 but not at a lower FDR threshold^12,13^. The high degree of replication between our eQTLs and a large published eQTL study^23^ (*π*_1_ = 0.96) and the relatively high internal replication rate for our sb-pQTLs (*π*_1_ = 0.52) lend confidence to our findings. Of note, the highest published internal replication rate for sb-eQTLs was *π*_1_ = 0.28 (ref. ^12^) and it was from breast tissue sb-eQTLs. Our findings are not entirely unexpected since we found generally low correlations (mean correlation of 0.11) between the mRNA and protein expression levels in 307 individuals with both transcriptomic and proteomic data for the 150 proteins with sb-pQTLs. These low correlations are consistent with the modest correlations between mRNA and protein levels in brain tissues observed in several studies^18–20,47^. Thus, differences in observed sb-pQTLs and sb-eQTLs are likely due to multiple factors ranging from technical (that is, differences in platforms for measuring mRNA and proteins) to biological (that is, differences in cell-type proportions or post-transcriptional gene regulations). Moreover, emerging evidence suggests that gonadal hormones and their receptors have pronounced effects on the expression and regulation of microRNAs^48–50^, which are important post-transcriptional regulators of gene expression, and on the translation of mRNAs^51^. Together, the more pronounced effects of sex on the genetic regulation of protein expression highlight an intriguing difference in what the transcriptome and proteome may reveal about sex-differentiated genetic control of gene expression in the human brain and should be further investigated.

In interpreting our findings we should take into consideration the limitations. Our studies were limited in power since each sex must be analyzed separately. There is also a limited number of sex-stratified GWAS that can make full use of the sex-specific pQTL data. Moreover, the available sex-stratified GWAS had much smaller sample sizes, and thus much lower power, compared with the joint GWAS (Supplementary Table 19). Collectively, these factors likely contribute to the relatively modest number of genes identified through integrating the sb-pQTLs with sex-stratified GWAS compared with traditional pQTL and GWAS integration in both sexes jointly. While it seems reasonable to speculate that gene-by-sex effects explain a small amount of the variance in the observed sex differences in brain traits, their utility lies in the mechanistic insights they provide, and larger sex-specific GWAS are needed to better gauge their contributions to disease. This limitation could be mitigated by standard reporting of sex-stratified GWAS results in addition to standard joint analysis results. Other limitations of this work include the profiling of brain proteomes and transcriptomes in individuals of European ancestry, which may potentially limit the generalizability of these findings to individuals of other ancestries. Additionally, focusing on older individuals may limit the generalizability of our findings to individuals across the age spectrum or to brain illnesses with earlier-onset age such as schizophrenia or bipolar disorder, although it likely reduces heterogeneity since all individuals can comfortably be assumed to have undergone menopause or andropause. Finally, while we found little evidence that our results of sex-biased genetic regulation were influenced by demographic factors correlated with sex, we caution that sex in this study should be thought of as biological sex and there are potentially many factors that differ between females and males. Thus, unmeasured environmental factors that are correlated with biological sex could potentially lead to the appearance of sex-biased gene expression or genetic regulation. Future studies should test gene-by-environment interactions to understand potential underlying mechanisms for sex differences in brain illnesses.

Strengths of our study include being the first study of the role of sex on protein abundance and its genetic regulation in the brain to the best of our knowledge. Second, it is the largest study of the role of sex in transcriptional expression, which enables us to compare the role of sex at the protein and transcript levels for a gene. Third, our study is based on a large dataset of well-characterized post-mortem brains that have undergone comprehensive proteomic and transcriptomic sequencing. Fourth, given the age of the donors, hormonal state was not likely to confound our results. Lastly, we presented here an invaluable resource of the largest brain pQTLs, sex-specific brain pQTLs and their concordance/discordance with sex-specific brain eQTLs, paving the way for future sex-aware studies of neuroscience and brain illnesses.

In conclusion, we found that biological sex has an influence on brain mRNA and protein expression and that its effects on genetic regulation of gene expression appeared more pronounced at the protein than mRNA level. Furthermore, we uncovered putative causal genes in brain traits and disease that have either sex-differentiated protein expression or sex-biased genetic regulation of protein expression. Finally, we provide a resource of human brain pQTLs, sb-pQTLs, eQTLs, sb-eQTLs and sex-specific causal proteins in psychiatric and neurologic disorders for the scientific community to study sex-aware mechanisms underlying brain illnesses.

## Methods

### Cohorts

Participants from the following studies donated post-mortem brain tissues for proteomic sequencing. The ROS and MAP are two community-based longitudinal clinical–pathologic studies of aging and AD^52^. All participants are organ donors, provide informed consent and sign an Anatomical Gift Act and repository consent to allow their data and biospecimens to be repurposed. An Institutional Review Board of Rush University Medical Center approved the studies.

The Arizona Study of Aging and Neurodegenerative Disorders, conducted by Banner Sun Health Research Institute (referred to as ‘Banner’ here), primarily recruits cognitively normal volunteers from the retirement communities and some participants with AD or Parkinson’s disease in Phoenix, AZ^53^. All participants receive annual standardized medical, cognitive and neurological assessments. Participants or their legal representatives signed an informed consent that was approved by the Banner Sun Health Research Institute Institutional Review Board allowing for brain donation and use of donated biospecimens for approved future research and genetic studies.

The Baltimore Longitudinal Study of Aging (BLSA) is a prospective study of aging in community-dwelling individuals. The BLSA study was approved by the Institutional Review Board and the National Institute on Aging. All BLSA participants provided written informed consent at each visit.

Post-mortem brain tissues also came from the Mount Sinai/JJ Peters VA Medical Center Brain Bank^54^. All donors or their representatives provided informed consent approved by the Institutional Review Boards of Mount Sinai School of Medicine and JJ Peters VA Medical Center. Donors with proteomic data had a mean age of 87 (range: 61–108). Donors with transcriptomic data had a mean age of 89 (range: 67–108).

No monetary compensation was provided to participants.

### Proteomic data

The proteomic sequencing of each dataset was preformed independently, and the methods for proteomic sequencing and quality control have been described in detail previously for all datasets^21,55^ except for the precuneus and middle frontal gyrus. All proteomic sequencing followed the same approach and was performed using isobaric tandem mass tag peptide labeling with peptides analyzed by liquid chromatography coupled to tandem mass spectrometry (MS). The proteomic sequencing for the precuneus and middle frontal gyrus are detailed here and are illustrative of the approach used for each dataset. Each sample was individually homogenized in urea lysis buffer to disrupt nucleic acids. Protein concentration was determined using the bicinchoninic acid method and frozen in aliquots at −80 °C. Protein mixture was digested overnight and diluted to obtain peptides. Peptides were labeled using TMTPro isobaric tags (Thermo Scientific). Subsequently, high pH fractionation was performed as described in research by Ping et al.^56^. All samples were then analyzed on the Evosep One system using the preprogrammed 21-min gradient as described by Bekker-Jensen et al.^57^. MS was performed with a high-field asymmetric waveform ion mobility spectrometry Pro equipped Orbitrap Eclipse (Thermo) in positive ion mode using data-dependent acquisition with 2-second top-speed cycles. Each cycle consisted of one full MS scan followed by as many MS/MS events that could fit within the given 2-second cycle time limit. All raw files were searched using Thermo’s Proteome Discoverer suite (v.2.4.1.15) with Sequest HT. The spectra were searched against a human uniprot database downloaded in August 2020 (86,395 target sequences). Percolator software implemented in the Thermo’s Proteome Discoverer suite was used to filter peptide spectral matches less than 1% FDR. Peptides were grouped using parsimony and unique peptides were used for protein-level quantitation. Reporter ions were quantified from MS2 scans using an integration tolerance of 20 ppm with the most confident centroid setting.

Quality control of the peptide sequencing was performed in each proteomics dataset separately and followed our previous approach^20,55^. All proteomic sequencing datasets included at least one global internal standard (GIS), but some included two GISs. For datasets with two GISs (that is, ROS/MAP, Banner and BLSA), proteins with abundance levels outside of the 95% confidence interval of the two GIS measurements within a batch were deemed not reliably measured and were thus excluded. For datasets with only one GIS per batch, this step was not performed. Next, proteins with missing values in more than 50% of the samples per dataset were removed. Protein abundance was normalized using the total abundance of all the proteins for that sample (to account for protein loading differences) and log_2_ transformed. To identify sample outliers, we performed iterative principal component analysis to remove samples with greater than four standard deviations from the mean of either the first or second principal component. Regression was used to estimate and remove the effects of batch, MS mode, age at death, post-mortem interval and clinical diagnosis from the proteomic profiles. To enable comparisons across datasets, a *Z*-score transformation was applied. For proteins with multiple isoforms, we selected the most abundant isoform for investigation. The three datasets from the dPFC were analyzed jointly, including a covariate for dataset, and all other datasets were analyzed separately. In total, there were six sets of proteomic data from six brain regions used in subsequent analyses (Supplementary Table 1).

### Genetic data

Genotypes were generated from blood or brain-derived DNA using microarrays (ROS/MAP and Banner) and/or whole genome sequencing (ROS/MAP and Mount Sinai Brain Bank) as described previously^54,55,58^. First, genotype quality control was performed on each dataset independently. Individuals with genotype missing rate > 5% were excluded and variants were excluded if they met any of the following criteria: genotype missing rate > 5%, minor allele frequency < 5%, Hardy–Weinberg equilibrium *P* < 5 × 10^−7^ and non-biallelic variants. Related individuals were identified using KING^59^ (v.2.2.2) and individuals who were second-degree or closer relatives were randomly removed. Individuals who were population outliers were identified and removed using EIGENSTRAT (v.6.1.4)^60^. All participants included in the analysis were of European ancestry. After initial genotype quality control, genotype data were merged, and a second round of population substructure and kinship analysis was applied to verify that the final dataset included only unrelated samples without population outliers. Lastly, EIGENSTRAT^60^ was used to derive genetic principal components and ten principal components were used as covariates in the quantitative trait locus analyses.

### Transcriptomic data

Transcriptomic profiling was performed as previously described in detail^61^. Briefly, RNA was extracted from post-mortem dPFC and sequenced on the Illumina HiSeq. Reads were aligned to a GRCh38 reference using STAR v.2.4 (ref. ^62^) and transcript level counts were computed. Transcripts with less than 1 count per million (CPM) for at least 50% of samples per clinical diagnosis of cognition (normal, AD or other), missing gene length or missing percentage guanine–cytosine content were removed. Two samples that were outliers based on principal component analysis of raw CPM values were removed. Raw counts for 15,582 genes from 632 individuals were available for analysis. We applied the variance stabilizing transformation (‘vst’ function) from the R package DESeq2 (ref. ^63^; v.1.26.0) to normalize for library size, reduce heteroskedasticity and transform to log_2_ CPM while protecting the effect of sex (by specifying design formula ~sex). Subsequently, we regressed out effects of batch, RNA integrity number, post-mortem interval, age and clinical diagnosis from the normalized transcriptomic data before performing the downstream analyses. Among these 621 transcriptomes, 307 were from donors of the proteomes above.

### Definition of sex

Sex was defined using genotyping data. In particular, biological sex was determined based on the heterozygosity rate across genetic variants located on the X chromosome in each donor using PLINK^64^. All donors included in our analyses had biological sex consistent with self-reported sex.

### Statistical analysis

#### SVA

We performed SVA using the SVA package^65^ (v.3.20.0) and the significant SVs were later used as covariates in the regression models to map QTLs and sb-QTLs. We derived 56 significant SVs from proteomic data and 33 significant SVs from transcriptomic data. For both proteomic and transcriptomic SV derivation, the effect of sex on gene expression was protected. In particular, in the SVA model, the primary variable of interest was sex and the expression matrix was the normalized protein expression levels. Of note, we already regressed out the effects of protein sequencing batch, post-mortem interval, study, age and cognitive diagnosis from the proteomic profile before using it as input in the SVA.

#### Sex differences in protein expression

We performed regression modeling with protein as the outcome, sex as the independent variable and 56 SVs as covariates in each brain region separately. To identify proteins with sex difference in expression across the different brain regions, we performed a meta-analysis following the MASH approach (MASHR v.0.2.38)^22^, which uses an empirical Bayesian approach to estimate correlations (using mash_estimate_corr_em) among these regions. The priors were effects in each brain region separately following the MASH approach^22^. We used both cov_pca and cov_flash from mashr to derive data-driven covariances. No statistical methods were used to predetermine sample sizes, but our sample size of 1,277 proteomes was larger than those reported in previous publications on sex differences in mRNA expression in brain^10,12^.

#### Sex differences in mRNA expression

The study of sex differences in mRNA expression was performed in an analogous fashion, as was done for protein using the transcriptomic count matrix and 33 SVs as covariates.

#### Sex differences in genetic regulation of protein abundance

The dPFC was used to investigate sex-differentiated genetic regulation of protein abundance because this brain region has the largest sample size with both proteomic and genetic data (*n* = 716). We examined proteins encoded by genes located on the 22 autosomes and X chromosome. For the latter, we coded the number of the minor allele as 0 or 2 for homozygous males and 0, 1, 2 for females. The window for QTL analysis was 500 kb up and downstream of the gene. To identify proteins with sb-pQTLs, we first performed a joint pQTL analysis in men and women combined using linear regression in PLINK, and sex-stratified pQTL analysis in men and women separately. Among the pQTLs identified at FDR < 0.05 in any of the above three analyses, we examined their genotype-by-sex interaction and declared those with a significant interaction at FDR < 0.05 as sb-pQTLs (Fig. 2). We performed FDR correction on all tested SNP–protein combinations. To identify index sb-pQTLs, we performed clumping with PLINK using the parameters of *r*^2^ of 0.50 and window size of 250 kb. The quantile–quantile plot for the *P* values of the genotype-by-sex term in the regression modeling for all the chromosomes (Extended Data Fig. 2a) and for the X chromosome (Extended Data Fig. 2b) showed no evidence of inflation, suggesting the underlying assumptions of the regression modeling were met, but this was not formally tested.

Likewise, to identify sb-QTLs among the genes that were profiled in both the proteomes and transcriptomes (*n* = 8,009 genes) for comparing sb-eQTLs to sb-pQTLs, we selected those genes and applied the same analysis framework as described above.

#### Sex differences in genetic regulation of mRNA expression

The study of sex differences in genetic regulation of mRNA expression was performed in an analogous fashion to sb-pQTLs, using the transcriptomic count matrix and 33 SVs as covariates.

#### Genomic site-type enrichment for the sb-pQTLs and sb-eQTLs

Variant annotation of the sb-QTL sites was performed with Bystro^66^. Fisher’s exact test was used to test for enrichment of different site types among the sb-QTL sites.

#### Internal replication (*π*_1_ statistics) of the sb-pQTLs

To determine the replication rate of sb-pQTLs, the ROS/MAP dPFC proteomic dataset was considered as the discovery dataset (*n* = 565) and the Banner dPFC proteomic dataset was considered the replication set (*n* = 151). In the discovery set, the independent sb-pQTLs were identified after clumping sb-pQTLs at FDR < 0.05 using the threshold of *r*^2^ < 0.5. Those results were compared with the sb-pQTLs of the replication dataset using the qvalue package v.2.22.0 (ref. ^67^) to estimate *π*_1._

#### Internal replication (*π*_1_ statistics) of the sb-eQTLs

The independent significant sb-pQTLs at FDR < 0.2 were tested for replication with the sb-eQTLs using the qvalue package in R to estimate *π*_1_.

#### Gene set enrichment analysis

Gene set enrichment analysis was performed using GO-Elite (v.1.2.5) for human species^68^, which included Biological Process^69^, Molecular Function^69^, Cellular Component^69^, WikiPathways^70^, KEGG^69^ and REACTOME^71^ databases. Fisher’s exact test and *Z-*scores were used to test for significant enrichment among the proteins of interest using a background of 10,198 assayed proteins. Multiple testing was addressed with the Benjamini–Hochberg FDR.

#### SuSiE regression

We performed multiple regression using the software package SuSIE^27^ and its default settings for the 12 causal genes with sb-pQTLs to examine whether there was more than one causal variant per gene.

#### Sex-specific PWAS

Sex-specific PWAS was performed following FUSION^24^ in males and females, respectively, using the sex-stratified GWAS results and sex-specific pQTL data we generated. We had access to the following sex-stratified GWAS for this analysis: major depression (*n* = 21,168 males and 27,372 females)^7^, bipolar disorder (*n* = 17,995 males and 21,554 females)^7^, schizophrenia (*n* = 32,152 males and 24,093 females)^7^, neuroticism (*n* = 137,880 males and 155,126 females; a personality trait that is prone to experiencing negative emotions)^28^, Parkinson’s disease (*n* = 110,616 males and 104,082 females)^29^ and AD proxy via family history of dementia (*n* = 141,897 males and 170,769 females)^28^ (Supplementary Table 19a).

First, we restricted the genotype data to the SNPs in the linkage disequilibrium reference panel provided with the FUSION^24^ package, which includes 1,190,321 SNPs from 1,000 Genome EUR samples, to minimize the influence of linkage disequilibrium on the analysis. Next, for each sex separately, SNP-based heritability for each protein was estimated. Proteins with SNP-based heritability *P* < 0.01 were declared heritable. Subsequently, for each heritable protein, we estimated the effect of a set of SNPs within a 500 kb window of the gene on its protein abundance, also referred to as the protein ‘weight’ for each sex separately. We applied the BLUP, LASSO, elastic net and BSLMM prediction models and kept the weights from the best-performing prediction model. Finally, we integrated the brain protein weights with each of the sex-specific GWAS summary statistics to perform the PWAS for each sex separately. The PWAS *Z-*score for each gene represents the combined effect of the protein and SNPs on the trait. The PWAS identified the *cis*-regulated proteins associated with the trait. We defined significant proteins as those with FDR *P* < 0.05 in each sex.

#### Sex-specific colocalization analysis

Sex-specific colocalization analysis was performed using COLOC^26^. Specifically, using the marginal association statistics, we estimated the posterior probability that a protein and trait share or do not share a genetic variant. We used the default previous values provided by COLOC, which were *p*_1_ = *p*_2_ = 10^−4^ and *p*_12_ = 10^−5^. We used the PP4 threshold of >75% to declare sharing a genetic variant.

#### Sex-biased causal genes and proteins

We defined genes and corresponding proteins identified in the PWAS and COLOC as genes and proteins consistent with a causal role or pleiotropy if they have FDR *P* < 0.05 in the PWAS and COLOC PP4 > 75% in the colocalization analysis. Moreover, we operationally defined causal genes/proteins as sex differentiated if they (1) have a PWAS FDR *P* < 0.05 and COLOC PP4 > 75% in females but a PWAS *P* > 0.05 or no PWAS *P* in males (since the proteins were not significantly heritable in males to run the PWAS) or (2) have a PWAS FDR *P* < 0.05 and COLOC PP4 > 75% in males but a PWAS *P* > 0.05 or no PWAS *P* in females.

### Reporting summary

Further information on research design is available in the Nature Portfolio Reporting Summary linked to this article.

## Online content

Any methods, additional references, Nature Portfolio reporting summaries, source data, extended data, supplementary information, acknowledgments, peer review information; details of author contributions and competing interests; and statements of data and code availability are available at 10.1038/s41591-023-02509-y.

## Supplementary information

Reporting Summary

Supplementary Tables Supplementary Tables 1–20.

## Data Availability

The data and results are deposited at Synapse at 10.7303/syn51150434. These data include raw, processed and normalized proteomic and transcriptomic data, sex-specific pQTLs, sex-specific eQTLs, and sex-specific protein weights from FUSION. These data are in whole or in part based on data obtained from the AMP-AD Knowledge Portal (https://adknowledgeportal.synapse.org/Explore/Programs/DetailsPage?Program=AMP-AD). The AD Knowledge Portal is a platform for accessing data, analyses and tools generated by the Accelerating Medicines Partnership (AMP-AD) Target Discovery Program and other National Institute on Aging (NIA)-supported programs to enable open-science practices and accelerate translational learning. The data, analyses and tools are shared early in the research cycle without a publication embargo on secondary use. Data are available for general research use according to the following requirements for data access and data attribution (https://adknowledgeportal.org/DataAccess/Instructions). The following databases for gene set enrichment analyses were used: Molecular Signatures Database (https://www.gsea-msigdb.org/gsea/msigdb/index.jsp); WikiPathways (https://www.wikipathways.org); KEGG pathway (https://www.genome.jp/kegg/pathway.html); and Reactome (https://reactome.org).

## Code availability

In-house pipelines and scripts used for this work are available at https://github.com/wingolab-org/role_of_sex_in_brain_expression.
