# Supplementary material for: Sex differences in brain protein expression and disease
Source: Nat Med. 2023 Aug 31;29(9):2224–32. doi: 10.1038/s41591-023-02509-y (PMC10504083; doi:10.1038/s41591-023-02509-y)
Supplement: Supplementary file 1 — Reporting Summary [file 41591_2023_2509_MOESM1_ESM.pdf]

nature portfolio

Corresponding author(s): Aliza P. Wingo, Thomas S. Wingo

Last updated by author(s): 7/3/2023

Reporting Summary

Nature Portfolio wishes to improve the reproducibility of the work that we publish. This form provides structure for consistency and transparency in reporting. For further information on Nature Portfolio policies, see our Editorial Policies and the Editorial Policy Checklist.

Statistics

For all statistical analyses, confirm that the following items are present in the figure legend, table legend, main text, or Methods section.

n/a	Confirmed	
☐	☒	The exact sample size (*n*) for each experimental group/condition, given as a discrete number and unit of measurement
☐	☒	A statement on whether measurements were taken from distinct samples or whether the same sample was measured repeatedly
☐	☒	The statistical test(s) used AND whether they are one- or two-sided<br>*Only common tests should be described solely by name; describe more complex techniques in the Methods section.*
☐	☒	A description of all covariates tested
☐	☒	A description of any assumptions or corrections, such as tests of normality and adjustment for multiple comparisons
☐	☒	A full description of the statistical parameters including central tendency (e.g. means) or other basic estimates (e.g. regression coefficient) AND variation (e.g. standard deviation) or associated estimates of uncertainty (e.g. confidence intervals)
☐	☒	For null hypothesis testing, the test statistic (e.g. *F*, *t*, *r*) with confidence intervals, effect sizes, degrees of freedom and *P* value noted<br>*Give P values as exact values whenever suitable.*
☐	☒	For Bayesian analysis, information on the choice of priors and Markov chain Monte Carlo settings
☒	☐	For hierarchical and complex designs, identification of the appropriate level for tests and full reporting of outcomes
☒	☐	Estimates of effect sizes (e.g. Cohen's *d*, Pearson's *r*), indicating how they were calculated

Our web collection on statistics for biologists contains articles on many of the points above.

Software and code

Policy information about availability of computer code

Data collection	A liquid chromatography coupled to tandem mass spectrometer (Thermo Scientific) was used to collect raw proteomic files using built-in Xcaliber software (https://www.thermofisher.com/order/catalog/product/OPTON-30965). Genetic data were generated using microarrays or whole genome sequencing. Transcriptomic data were profiled using Illumina HiSeq.
Data analysis	Proteome Discoverer suite (version 2.4.1, Thermo Scientific) was used to analyze proteomic raw files. Percolator was part of this suite. R version 3.5.1 (https://www.r-project.org/) was used for regression modeling. SVA v3.20.0 (https://www.rdocumentation.org/packages/sva/versions/3.20.0) was used for surrogate variable analysis. Plink version v1.90 beta (https://www.cog-genomics.org/plink/1.9/) was used for genetic analysis. KING version 2.2.2 (https://www.kingrelatedness.com/history.shtml) was used to identify cryptic relatedness. EIGENSTRAT v6.1.4 (https://github.com/DReichLab/EIG/tree/master/EIGENSTRAT) was used to derive genetic principal components. MASHR v0.2.38 (https://github.com/stephenslab/mashr) was used for meta-analysis. STAR v2.4 (https://github.com/alexdobin/STAR) was used for alignment of transcriptomic data. R package DESeq2 v.1.26.0 (https://bioconductor.org/packages/release/bioc/html/DESeq2.html) was used to normalize transcriptomic data. The qvalue package version 2.22.0 (https://www.bioconductor.org/packages/release/bioc/html/qvalue.html) was used for replication analysis. GO-Elite version 1.2.5 (http://www.genmapp.org/go_elite/) was used for gene set enrichment analysis. Bystro (https://github.com/akotlar/bystro/blob/b10/INSTALL.md) was used for variant annotation of sb-pQTL. FUSION (http://gusevlab.org/projects/fusion) was used to perform PWAS in each sex separately. COLOC v5.0.0.9002 (https://chr1swallace.github.io/coloc/) was used for colocalization analysis in each sex separately. SusieR (https://stephenslab.github.io/susieR/index.html) was used to examine possibility of multiple causal variants for a gene. In-house pipelines and scripts used for this work are available at https://github.com/wingolab-org/role_of_sex_in_brain_expression.

For manuscripts utilizing custom algorithms or software that are central to the research but not yet described in published literature, software must be made available to editors and reviewers. We strongly encourage code deposition in a community repository (e.g. GitHub). See the Nature Portfolio guidelines for submitting code & software for further information.

Data

Policy information about availability of data

All manuscripts must include a data availability statement. This statement should provide the following information, where applicable:

- Accession codes, unique identifiers, or web links for publicly available datasets
- A description of any restrictions on data availability
- For clinical datasets or third party data, please ensure that the statement adheres to our policy

The data and results are available at DOI: https://doi.org/10.7303/syn51150434. These data include raw, processed, and normalized proteomic and transcriptomic data, sex-specific pQTLs, sex-specific eQTLs, and sex-specific protein weights from FUSION. These data are in whole or in part based on data obtained from the AMP-AD Knowledge Portal. The AD Knowledge Portal is a platform for accessing data, analyses, and tools generated by the Accelerating Medicines Partnership (AMP-AD) Target Discovery Program and other National Institute on Aging (NIA)-supported programs to enable open-science practices and accelerate translational learning. Data are available for general research use according to the following requirements for data access and data attribution (https://adknowledgeportal.org/DataAccess/Instructions). We also used the following databases for gene set enrichment analyses: Molecular Signatures Database (https://www.gsea-msigdb.org/gsea/msigdb/index.jsp); WikiPathways (https://www.wikipathways.org); KEGG pathway (https://www.genome.jp/kegg/pathway.html); Reactome (https://reactome.org).

Human research participants

Policy information about studies involving human research participants and Sex and Gender in Research.

Reporting on sex and gender	The manuscript refers to sex in the biological sense of the word. Sex was determined based on the genotyping of the X chromosome. Particularly, biological sex was determined based on the heterozygosity rate across genetic variants located on the X chromosome in each donor using Plink. As part of genotyping quality control, we checked the agreement between self reported gender and genetic sex and they agreed for all subjects included in our analyses.
Population characteristics	The ROS/MAP and Banner post-mortem brain donors were recruited from the community. The Mt Sinai Brain Bank donors were from the Mt. Sinai and JJ Peters VA Medical Center Brain Bank. Mean age of donors ranged from 82 to 91. Approximately 63% of donors were females.
Recruitment	Participants were recruited from the community by the Rush Memory and Aging Project, Religious Order Study, and Arizona Study of Aging and Neurodegenerative Disorders. We are not aware of any self-selection bias or other bias that may affect the study besides the focus on participants of retirement age (65 or above). All research participants and post-mortem brain donors provided informed consent approved by the Institutional Review Boards of Rush University Medical Center, Banner Sun Health Research Institute, National Institute on Aging, Mount Sinai School of Medicine, and JJ Peters VA Medical Center, respectively.
Ethics oversight	All the studies (ROS/MAP; Banner, Mt. Sinai Brain Bank) received approval for the studies from an Institutional Review Board at their academic affiliates.

Note that full information on the approval of the study protocol must also be provided in the manuscript.

Field-specific reporting

Please select the one below that is the best fit for your research. If you are not sure, read the appropriate sections before making your selection.

☒ Life sciences ☐ Behavioural & social sciences ☐ Ecological, evolutionary & environmental sciences

For a reference copy of the document with all sections, see nature.com/documents/nr-reporting-summary-flat.pdf

Life sciences study design

All studies must disclose on these points even when the disclosure is negative.

Sample size	No sample size calculations were used. All available samples with brain proteomic, genetic, and phenotypic data were used for the analysis.
Data exclusions	Outlier samples were removed in the quality control step of proteomic data. This was done through an iterative process of detecting outliers by principal component analysis of the proteomic data and excluding all individuals who were greater than 4 standard deviations from the mean of the first two principal components. Then we included individuals with both proteomic and genome-wide genotyping data for the analyses.
Replication	Internal replication of the sb-pQTLs was performed using the π1 statistics and found to be 0.53, which is higher than published replication rate for sb-eQTLs.
Randomization	For proteomic sequencing, samples were randomized by age, sex, PMI, clinical diagnosis, and pathologies into batches of 8 samples to minimize the batch effects.

Blinding	The individuals preparing samples for proteomic sequencing were blinded to phenotypic information.

Reporting for specific materials, systems and methods

We require information from authors about some types of materials, experimental systems and methods used in many studies. Here, indicate whether each material, system or method listed is relevant to your study. If you are not sure if a list item applies to your research, read the appropriate section before selecting a response.

Materials & experimental systems

n/a	Involved in the study
☒	☐ Antibodies
☒	☐ Eukaryotic cell lines
☒	☐ Palaeontology and archaeology
☒	☐ Animals and other organisms
☒	☐ Clinical data
☒	☐ Dual use research of concern

Methods

n/a	Involved in the study
☒	☐ ChIP-seq
☒	☐ Flow cytometry
☒	☐ MRI-based neuroimaging
